# Expression and Function of IL-33/ST2 Axis in the Central Nervous System Under Normal and Diseased Conditions

**DOI:** 10.3389/fimmu.2018.02596

**Published:** 2018-11-20

**Authors:** Karen Fairlie-Clarke, Mark Barbour, Chelsey Wilson, Shehla U. Hridi, Debbie Allan, Hui-Rong Jiang

**Affiliations:** Strathclyde Institute of Pharmacy and Biomedical Sciences, University of Strathclyde, Glasgow, United Kingdom

**Keywords:** IL-33, ST2, central nervous system, expression, neurological diseases

## Abstract

Interleukin-33 (IL-33) is a well-recognized immunomodulatory cytokine which plays critical roles in tissue function and immune-mediated diseases. The abundant expression of IL-33 in brain and spinal cord prompted many scientists to explore its unique role in the central nervous system (CNS) under physiological and pathological conditions. Indeed emerging evidence from over a decade's research suggests that IL-33 acts as one of the key molecular signaling cues coordinating the network between the immune and CNS systems, particularly during the development of neurological diseases. Here, we highlight the recent advances in our knowledge regarding the distribution and cellular localization of IL-33 and its receptor ST2 in specific CNS regions, and more importantly the key roles IL-33/ST2 signaling pathway play in CNS function under normal and diseased conditions.

## Introduction

The active and extensive communication between the immune and central nervous system (CNS) is essential in maintaining homeostasis of the CNS and mediating the pathogenesis of neurological diseases. Over recent decades, rapid development in the area suggests many immune cytokines play pivotal roles facilitating this complex neuroimmune crosstalk, and interleukin-33 (IL-33) is one such key cytokine.

IL-33, a member of IL-1 cytokine family, was first identified in 2005 (1) as the ligand for ST2 (also known as IL-1RL1) (2). Extensive research in the following years has shown that full length IL-33 is bioactive and can be released by living cells (3), or by necrotic cells following tissue damage acting as an endogenous danger signal or alarmin [reviewed previously (4, 5)]. In apoptotic cells, IL-33 is inactivated by released caspases (6). IL-33 binds to ST2 and then recruits IL-1 receptor accessory protein (IL-1RAcP), which leads to the activation of MyD88 and NF-κB signaling pathway (7). Soluble ST2 (sST2), a decoy receptor for IL-33, binds to IL-33, and blocks its function. While IL-33 is expressed by many types of cells, in particular stromal and tissue barrier cells and some innate immune cells, ST2 is expressed by various immune cells including T cells, B cells, macrophages, dendritic cells, and innate lymphoid cells (7). The IL-33/ST2 signaling pathway has pleitropic functions in a range of infectious and inflammatory diseases often with dual roles, mediating both pathological immune responses and tissue repair (4, 8–11).

Remarkably IL-33 is expressed constitutively within the brain and spinal cord tissues. Around 33% of isolated brain cells of naïve mouse are IL-33 positive (12) and its mRNA expression level is higher than any other tissues and organs tested (1). These findings together with the recent evidence of ST2 expression by CNS resident cells (13, 14) indicate a unique role for the IL-33/ST2 signaling pathway within the CNS. Indeed accumulating evidence in recent years suggests that IL-33 mediates the interaction between immune, endothelial and CNS resident cells and plays a key role in the development and homeostasis of the CNS. IL-33 is also prominently involved in the neuroinflammation of many neurological diseases such as Alzheimer's disease (AD) and multiple sclerosis (MS) through action mechanisms beyond immunomodulation (15, 16).

This paper will summarize the current findings of the expression of IL-33 and ST2 in different regions and cells of the CNS. This is an area which remains poorly characterized and sometimes controversial, however it constitutes a key step toward our understanding of the function of IL-33/ST2 axis in CNS. We will then discuss the functional implications of the IL-33/ST2 signaling pathway in the CNS compartment under normal and diseased conditions.

## Diverse expression of IL-33 and its receptor ST2 in CNS regions and cells

### IL-33 expression

**Figure 1:**
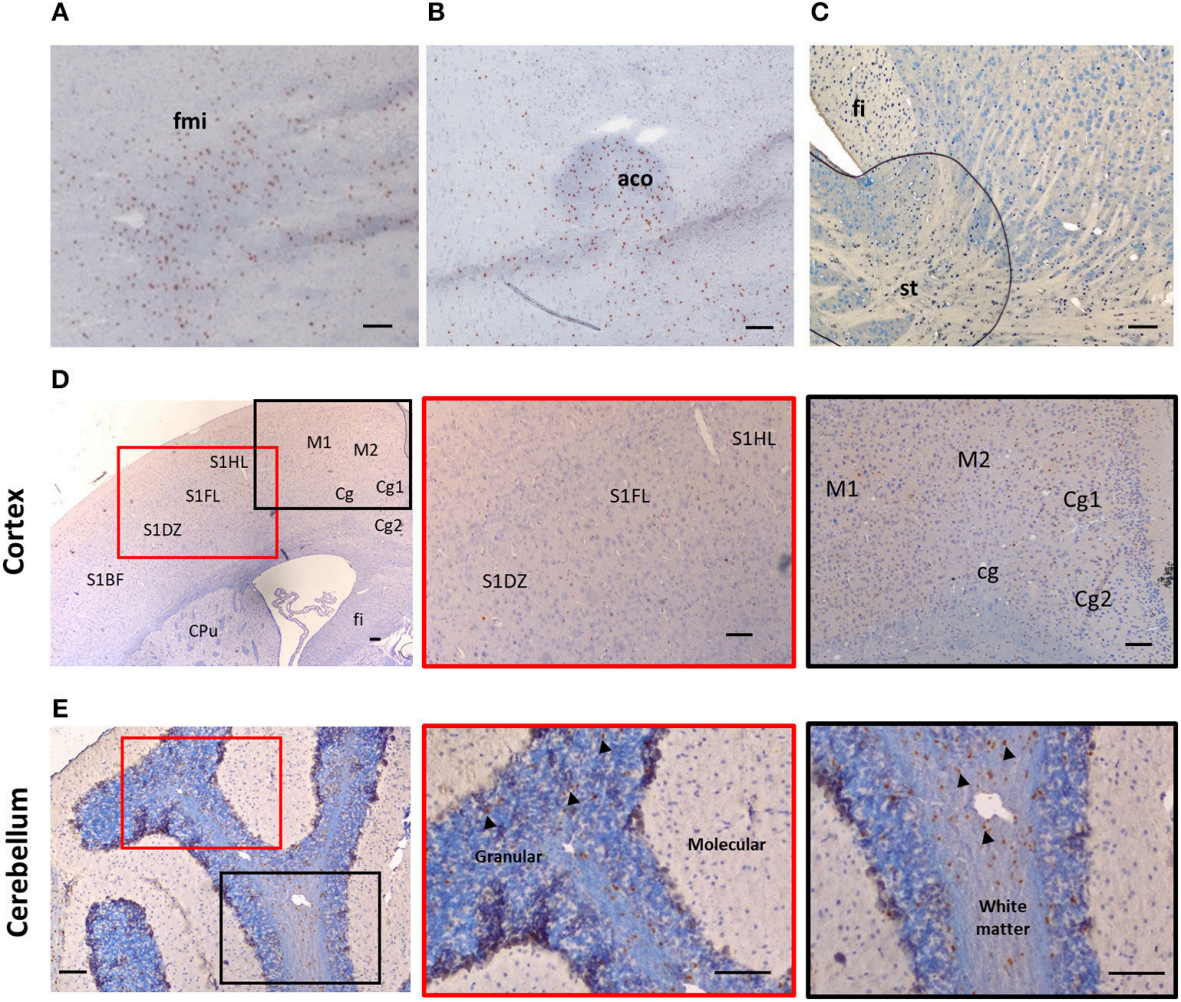
Expression of IL-33 in several brain regions. Brains from C57BL/6 mice were sectioned coronally and immunohistochemically stained for the expression of IL-33 (red/brown) and counterstained with haematoxylin. **(A–C)** IL-33 is predominantly expressed in several white matter rich regions including **(A)** anterior forceps of corpus collosum (fmi); **(B)** anterior commissure (aco) and **(C)** the hippocampal fringe (fi) and stria terminalus (st). **(D)** Within the cortex, IL-33 is expressed at low levels in the somatosensory (S1) and motor cortex regions (M1/M2). **(E)** In the cerebellum, IL-33 is expressed through the granular, and white matter regions with minimal expression within the molecular layer. Arrowheads indicate IL-33^+^ cells. CPu, striatum; Cg, cingulate cortex. Scale bars represent 100 μm.

Despite suggestions from Wicher et al. that IL-33 is only expressed in CNS during late embryogenesis and becoming absent in adult brain (17), others show that its expression is increased during postnatal development (16, 18). IL-33 is also constitutively highly expressed in adult CNS tissues in both human and mouse (13, 16, 19, 20). Interestingly the levels of IL-33 expression across brain and spinal cord regions are not uniform and constant, which potentially reflects specific functions of the IL-33/ST2 signaling pathway in region-associated neuronal activities and disorders. IL-33 expression is particularly abundant in white matter areas such as: corpus callosum (CC) (12, 21) and the anterior forceps of the CC (fmi) (Figure 1A), anterior commissure (aco) (Figure 1B), hippocampal fringe (fi) and the stria terminalus (st) (Figure 1C) (21, 22). IL-33 expression is also distributed throughout the olfactory cortex as well as scattered expression in the somatosensory (S1) and motor cortex (M1/M2; Figure 1D). While the hippocampus is another region which is often affected during CNS inflammatory diseases, in particular AD, a disease accompanied by cognition and memory impairments (23), hippocampal expression of IL-33 is surprisingly low (21). Within the cerebellum, IL-33 expressing cells are scattered through the granular, and white matter layers with no expression detected in the molecular layer (Figure 1E).

**Figure 2:**
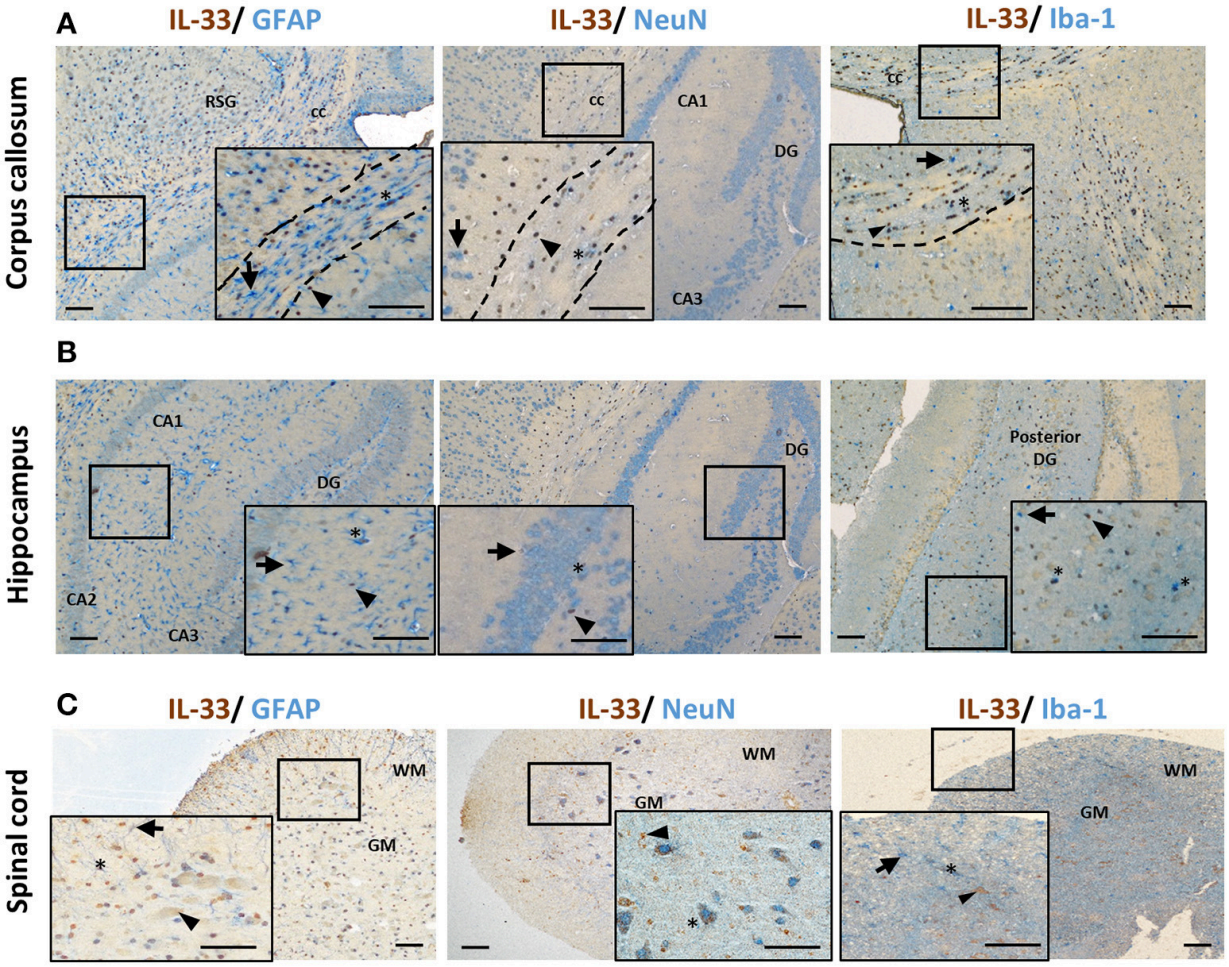
IL-33 expression by CNS resident cells in mouse brain and spinal cord. Tissues from C57BL/6 mice were sectioned and immunohistochemically stained for the expression of IL-33 (brown), and astrocytes (GFAP), neurons (NeuN) and microglia (Iba-1) (all blue) within the **(A)** corpus callosum and **(B)** hippocampus and IL-33 co-localization assessed. RSG, retrosplenial granular cortex; CC, corpus callosum; DG, dentate gyrus; CA1-3, hippocampus. **(C)** In spinal cord, the majority of IL-33 expressing cells are localized to the GM, with lower levels present in the WM. IL-33 is expressed by GFAP^+^ astrocytes in both GM and WM regions with lower level co-localization with neurons and microglia in the GM and, GM/WM, respectively. Arrowheads indicate IL-33^+^ cells, arrows indicate NeuN, GFAP or Iba-1^+^ cells and asterisks indicate double positive cells. GM, gray matter; WM, white matter. Scale bars represent 100 μm.

Co-localization of IL-33 with various CNS resident cells has been reported across brain regions (Table 1). Within the CC, IL-33 is predominantly expressed by astrocytes (not all though) in murine tissues using GFAP (Figure 2A) or S-100 antibodies (27). High level of co-expression with Oligo2^+^ oligodendrocytes is also shown within this region (21), which is enhanced by Poly-IC treatment after gliotoxic injury and therefore promotes oligodendrocyte progenitor cell (OPC) maturation and myelin production (27). Whether IL-33 is expressed by microglia and neuron cells is less clear and remains controversial (Table 1). IL-33 colocalisation with Iba-1^+^ microglia and neuronal somas (NeuN^+^) (Figure 2A) in CC region is evident however at much lower levels. In the hippocampus, the low level of IL-33 expression appears to co-localize with GFAP^+^ astrocytes predominantly within the stratum oriens and stratum radiatum layers of the CA1, CA2, and CA3 region as well as molecular layer of the dentate gyrus (DG; Figure 2B). Consistent with previous findings (21), low level neuronal expression of IL-33 is observed in the granular layer (Figure 2B) within the DG, while low number of IL-33-expressing microglia was also observed in the polymorph layer of the DG (Figure 2B) (21).

**Table 1:** Expression of IL-33 in central nervous system cells.

**CNS Tissue**	**Neuron (NeuN Ab or stated)**	**Astrocyte (GFAP Ab or stated)**	**Microglia (Iba-1 Ab or stated)**	**Oligodendrocytes (Olig 2 Ab or stated)**	**References**
Mouse mixed glia culture: *Il33* mRNA	ND	√	ND	NT	(14)
Mouse mixed glial culture	NT	√	NT	NT	(24)
Mouse mixed glial culture	NT	√	NT	√	(17)
Mouse mixed glial culture	NT	√	NT	√	(25)
Mouse P9 brain	ND in most MAP-2+ cells	√	NT	√	(17)
Rat brain	√	√	ND	NT	(26)
Rat brain	NT	√√ (S-100)	Infrequent (CD68)	√	(27)
MS patient brain	NT	√	NT	NT	(19)
Healthy and MS brain	√ (SMI-31)	√	√	√ (CA-II)	(13)
Mouse brain	ND (MAP-2 and DCX)	√	ND	√	(25)
Mouse brain	√ (ventral dentate gyrus)	√ (S-100β)	ND	√	(21)
Mouse brain	ND	√	ND	√ (CC-1, not NG2)	(28)
Mouse brain	NT	√	NT	NT	(18)
Mouse thalamus and spinal cord	NT	√ (ALDH1L1)	NT	√ (CC-1)	(16)
Mouse spinal cord	√	√	NT	NT	(29)
Mouse spinal dorsal horn	ND	√	ND (CD11b)	NT	(30)
Mouse spinal cord	ND	√	ND	NT	(31)
Mouse spinal cord	√	√	ND (CD11b)	NT	(32)
Mouse spinal cord	ND	√	ND (CD11b)	NT	(33)
Mouse spinal cord	Minimal to none	√ (ALDH1L1)	Minimal to none	√ (Olig2+CC-1)	(12)
Mouse spinal cord	√	√	√	√	(34)
Rat spinal cord	√	√	ND (OX-42)	√	(35)

*Expression of Il33 mRNA was performed using PCR while protein expression was studied using immunohistochemical staining and other methods. √, positive expression of IL-33; ND, not detectable; NT in gray square, not tested. Ab, antibody; MS, multiple sclerosis*.

The spinal cord is made up of bundles of nerve fibers and forms an important part of the CNS. Currently it is not clear whether IL-33/ST2 exhibit different roles in the spinal cord compared to brain. Data of IL-33 expression in the spinal cord has been primarily documented in animal studies (Figure 2C) (16, 29, 30, 34). In contrast to its abundant expression in the white matter region in brain (12), IL-33 immune reactivity is prominent in spinal cord gray matter and significantly lower in the white matter (29). This suggests potentially different functions of IL-33 in brain and spinal cord. Similar to findings in the brain, IL-33 is shown to be expressed by oligodendrocytes (34) and astrocytes (29, 30) (Figure 2C). In a recent study, Vainchtein et al. identified astrocytes as the primary source of local IL-33 in both brain and spinal cord using two IL-33 reporter mouse models (16). Further evidence also suggests that IL-33 is expressed by a limited number of NeuN^+^ neurons and Iba1^+^ microglia cells (Figure 2C), similar to its expression in brain.

### ST2 expression

Extracellular IL-33 exerts its effect through binding to its receptor which consists of a heterodimer between ST2 and IL-1RAcP (4). Although the expression pattern and cellular distribution of ST2 in CNS remains understudied and somewhat disputed, there has been some exciting progress in recent years identifying IL-33 target cells. This has provided new insights into the functional mechanisms of the signaling pathway in CNS.

**Figure 3:**
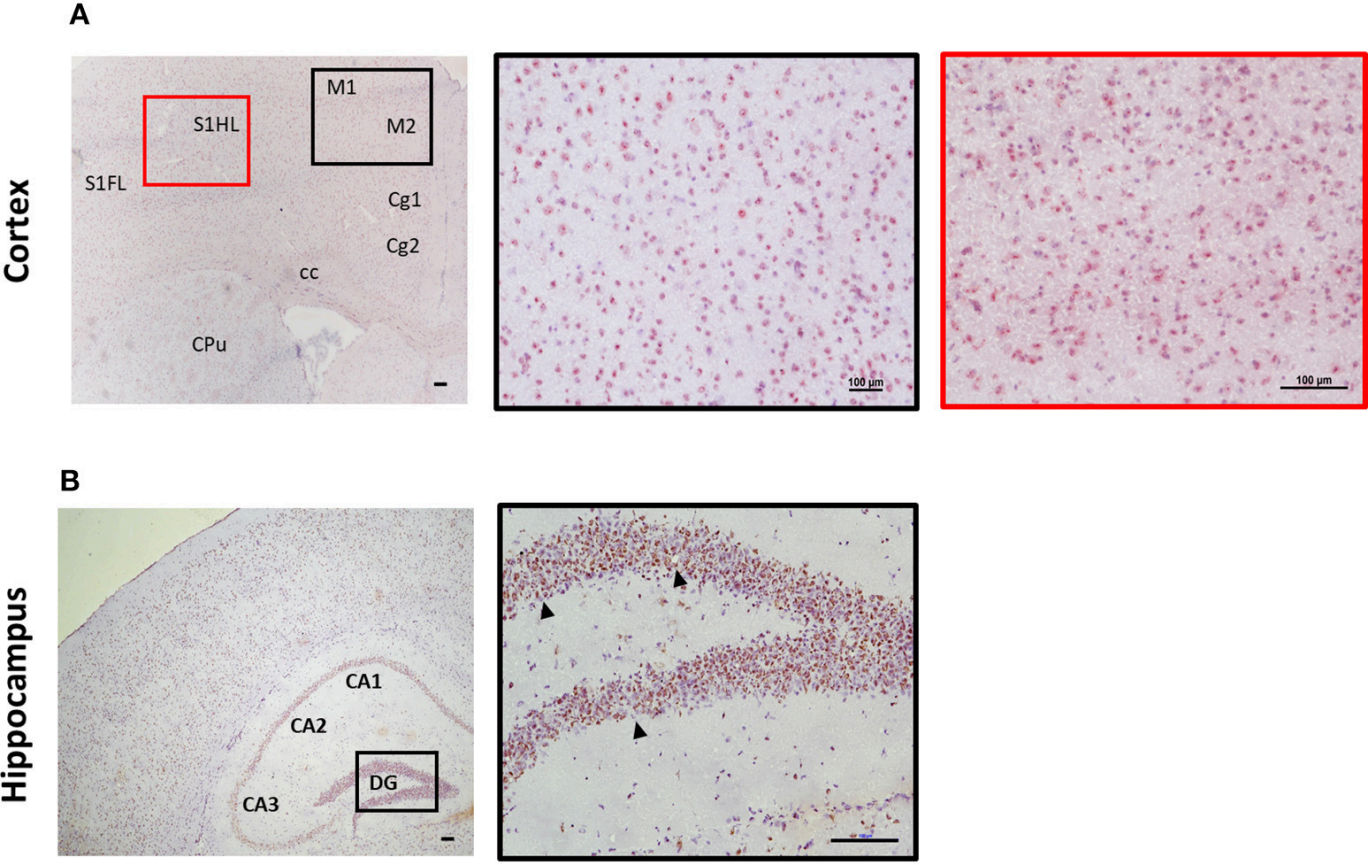
Expression of ST2 in mouse brain regions. Brains from C57BL/6 mice were sectioned coronally and stained for the expression of ST2 (red/brown) and counterstained with haematoxylin. **(A)** Within the cortex, ST2 was expressed at high levels in the somatosensory (S1) and motor cortex regions (M1/M2). **(B)** ST2 was also highly expressed throughout the hippocampus (CA1–CA3) including the dentate gyrus (DG). Arrowheads indicate ST2^+^ cells. CPu, striatum; Cg, cingulate cortex. Scale bars represent 100 μm.

Expression of ST2 in the brain appears to mirror IL-33 expression in regard to distribution pattern. This includes regions such as the olfactory bulb, hippocampus, and hippocampal fringe; anterior commissure; CC as well as a number of other regions throughout the cortex, in particular within the somatosensory regions (S1FL/HL; Figure 3A). ST2 expression within the cerebellum is distinct with low levels in the granular and white matter layer, and likely on Purkinje cells (personal observation), a class of GABAergic neurons located in the cerebellum and cerebellar nuclei which play a fundamental role in controlling motor movement. Whether IL-33/ST2 in cerebellum region relates to motor movement activities is not clear. In contrast to the low level of IL-33 expression observed in the hippocampus (Figure 2B), ST2 is highly expressed throughout all regions which include CA1-CA3 and DG (Figure 3B).

**Figure 4:**
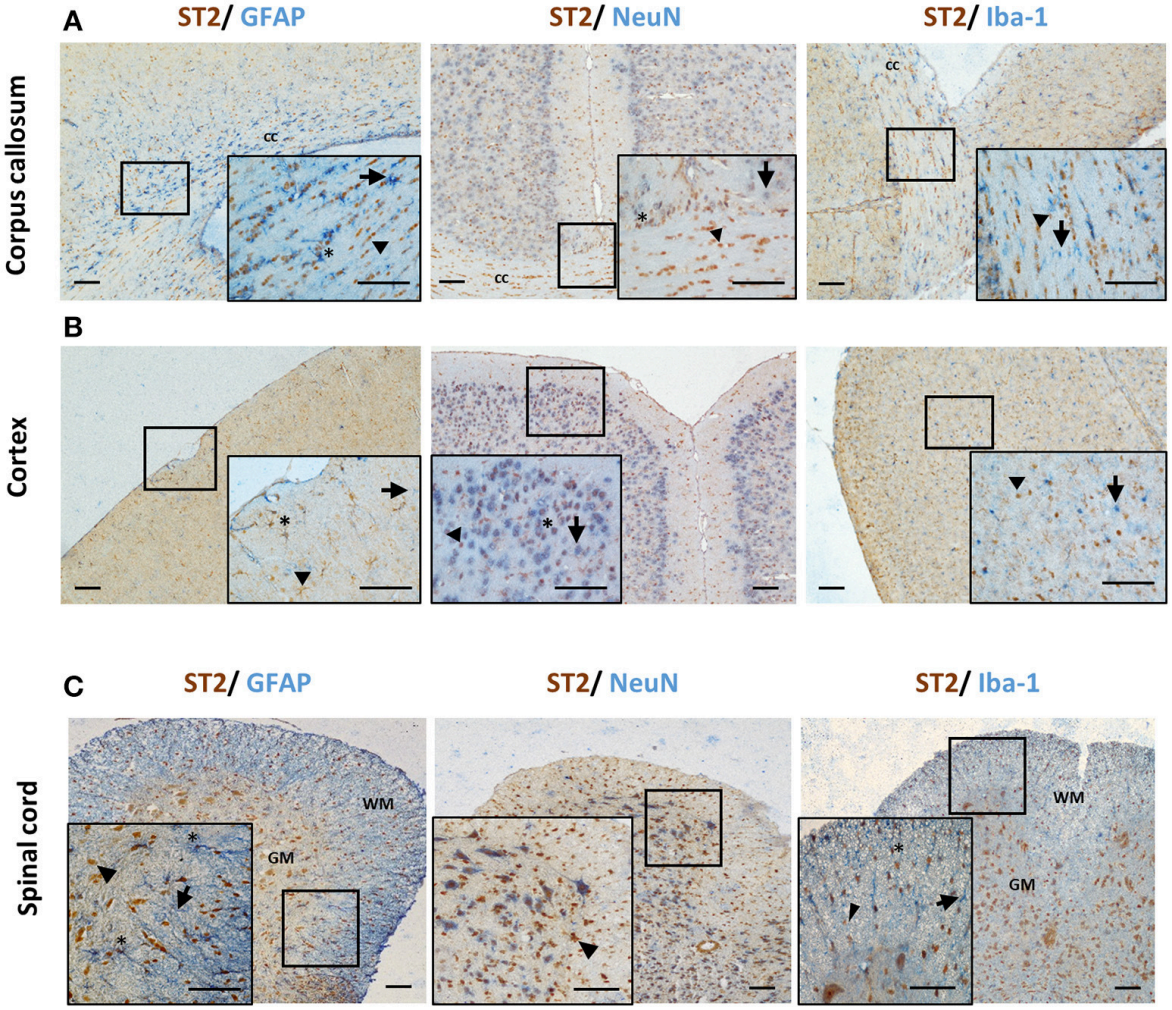
ST2 expression by CNS resident cells in mouse brain and spinal cord. Tissues from C57BL/6 mice were sectioned and stained for the expression of ST2 (brown) and astrocytes (GFAP), neurons (NeuN) and microglia (Iba-1) (all blue) within the **(A)** corpus callosum and **(B)** hippocampus and ST2 co-localization assessed. The majority of ST2 expressing cells co-localized with GFAP^+^ astrocytes while low level co-localization with neurons and microglia was observed in both regions. **(C)** In spinal cord, the majority of ST2 expressing cells were localized to the GM, with lower levels present in the WM. ST2 was expressed by NeuN^+^ neurons in both GM and WM regions with lower level co-localization with astroyctes and microglia. Arrowheads indicate ST2^+^ cells, arrows indicate NeuN, GFAP or Iba-1^+^ cells and asterisks indicate double positive cells. cc, corpus callosum; GM, gray matter; WM, white matter. Scale bars represent 100 μm.

Different CNS cells have been proposed as IL-33 target cells (Table 2). *St2* mRNA was initially identified in cultured murine astrocytes and microglia (14). However only microglia, not astrocytes, neurons or any other CNS cells, flow-sorted from thalamus region tissues are confirmed to express *St2* (16). Extensive staining in naïve mouse brain tissue demonstrates that within the CC region, ST2 is surprisingly expressed by some astrocytes, with lower expression level observed in neuron cell bodies and microglia (Figure 4A). Prominent but not complete neuronal co-localization is also seen in the cortex (Figure 4B), indicating the presence of ST2 on other CNS cell types such as GFAP^+^ astrocytes (Figure 4B). The expression of ST2 on astrocytes close to the endothelial layer of the cortex is interesting, as these cells could be involved in the astrocytic interactions with endothelial cells and pericytes making up the blood brain barrier (BBB) (38). IL-33 may interact with the BBB via these ST2^+^ astrocytes as reported in an experimental cerebral malaria (ECM) model (39). Furthermore, ST2 expression in various brain regions changes under different conditions, e.g., the level is upregulated in the lesion of ischemic brain (28).

**Table 2:** Expression of IL-33 receptor ST2 in central nervous system cells.

**Tissue or cells**	**Neuron (NeuN Ab or stated)**	**Astrocyte (GFAP Ab or stated)**	**Microglia (Ab tested)**	**Oligodendrocytes (Olig 2 Ab or stated)**	**References**
Mouse mixed glia culture: *St2* and *IL-1RAcP* mRNA	√ (*IL-1RAcP* only)	√	√	NT	(14)
Mouse mixed glia culture: *St2* mRNA	NT	√	√	NT	(12)
Rat myelinating co-culture	√	ND	NT	√ (O4)	(13)
MS patient brain	√ (SMI-31)	NT	NT	√ (CA-II)	(13)
Mouse brain	ND (by FC)	√ (GLAST) (by FC)	√ (Iba-1)	ND (by FC)	(28)
Mouse thalamus and spinal cord flow sorted cells (*St2* mRNA)	ND	ND	√	NT	(16)
Mouse spinal cord	√	ND	NT	NT	(29)
Mouse spinal cord	√	√	ND (CD11b)	NT	(36)
Mouse dorsal root ganglia	√ (Nissi)	NT	NT	NT	(36)
Human DRG: *St2 and IL-1RAcP* mRNA	√	NT	NT	NT	(36)
Mouse spinal cord (EAE)	NT	√	NT	√ (O1)	(37)
Rat spinal cord	√	√	ND (OX-42)	√	(35)

*Expression of St2 mRNA was performed using PCR while protein expression was studied using immunohistochemical staining or other methods. √, positive expression of ST2; ND, not detectable; NT in gray square, not tested. Ab, antibody; EAE, experimental autoimmune encephalomyelitis; FC, flow cytometry; MS, multiple sclerosis; DRG, dorsal root ganglia*.

The expression of ST2 in the spinal cord was confirmed years ago (29) however it is yet to be agreed which CNS cell population is the target for IL-33. Findings from several groups have indicated that neurons (29, 33, 35), astrocytes (33, 35) and oligodendrocytes (35, 37) can all express ST2 in the spinal cord as also shown in Figure 4C. Neuronal expression of ST2 within the gray matter is particularly evident (Figure 4C) and its staining pattern is consistent with the morphology of neurons, which was confirmed by dual staining with NeuN antibody. Less is known about ST2 expression by spinal cord microglia cells (33, 35). Immunohistochemical staining of naïve mouse tissue confirms the expression of ST2 in some astrocytes and microglia (Figure 4C), albeit at far lower levels. In the inflammatory spinal cord of experimental autoimmune encephalomyelitis (EAE) mice, mRNA of *St2* is shown to be increased (29) and the upregulation is predominantly in the lesion area in the white matter (29, 37).

### Implications of IL-33 and ST2 expression in CNS

Thus, the current research evidence illustrates a complex pattern of IL-33 and ST2 expression in CNS tissue and cells. The variation of the expression levels in different regions of the CNS may indicate that the IL-33/ST2 signaling pathway has specific roles in different regions. For example, CC region is the largest commissure in the brain connecting both hemispheres homologous regions and providing interhemispheric communication (40). It is also involved in a number of CNS inflammatory diseases including MS (41, 42) and cerebral malaria (25). High levels of both IL-33 and ST2 positive cells in the CC may imply a role of the signaling pathway in these diseases. While it is surprising that IL-33 expression in hippocampus is low, high levels of ST2 expression in the region may indicate an important hippocampal response to IL-33 released from nearby tissues following injury/inflammation.

Numerous studies have substantiated that astrocytes and oligodendrocytes are the predominant source of IL-33 production, while its expression on neurons and microglia (Table 1) is less consistent and requires further investigation. Once produced, IL-33 acts through binding to ST2 receptor expressed by various cell populations including microglia, oligodendrocytes, astrocytes and neurons. The variation and disagreement about IL-33^+^ and ST2^+^ cells in CNS tissues between reports (Tables 1, 2) may be explained by the heterogeneity of each type of CNS cell. For example astrocytes differ in morphology, receptor expression, and function across different CNS regions, this has been reported in both mice and human (43–45). Furthermore, without doubt, the dynamic neurological and immunological changes within the tissue under various physiopathological conditions also contribute to the complex heterogeneity of CNS resident cells. This inevitably influences the expression of IL-33 and ST2 on these cells. IL-33 expression is increased in the CNS of MS patients (13) but the level is decreased in AD (46). ST2 expression is significantly increased in the spinal cord lesions composed of infiltrating immune cells in EAE mice (37), and in microglia which are essential in mitigating the severity of ischemic lesions in mice (28). Gadani et al. even discovered a flip of the expression levels of *St2* gene between CD11b^+^ microglia and CD11b^−^ astrocyte cell populations before and after injury (12). It is also important to note the different methods used to determine the molecule expression in tissues, e.g., *Il-33* reporter mice (16, 25) or immunohistochemical staining. Although there is variation in the quality of the IL-33 and ST2 antibodies used in different experiments, IL-33^−/−^ mice (21) or specific blocking peptides (36) are used in some but not all studies to verify the specificity of the antibodies. Improvement in the standards for antibody validation is therefore essential in the future.

Taken together, the dynamic expression of IL-33 and its receptor ST2 within the CNS regions and cells indicates a complex network of communication between the immune and CNS resident cells. This underscores the fundamental role endogenous IL-33/ST2 signaling pathway plays in the CNS under physiological and pathological conditions.

## Role of IL-33/ST2 axis in CNS development and function

Despite recent effort, whether IL-33 has detrimental or beneficial effects on the growth and function of neurons is still undetermined. 10 ng/ml of recombinant IL-33 (rIL-33) in a mouse mixed glia cell culture or pure neuronal culture decreases neuronal number together with the loss of neuritis-like appearance when compared with untreated control neurons (47). However 25 or 100 ng/ml of rIL-33 has no significant impact on the growth or viability of neurons and axonal densities in a rat myelinating culture system (13). Similarly, there is contradictory evidence regarding the role of IL-33/ST2 axis in CNS myelination. Treatment with rIL-33 promotes the differentiation and maturation of rat OPCs cultured *in vitro* (27), but fails to improve axonal myelination in a rat myelinating culture as the treatment significantly reduces the proportion of myelinated axons (13). It is worth noting that this inhibitory effect on myelination is not observed in a mouse myelinating culture system (13). This may indicate an important species-specific difference or indeed culture condition difference resulting in the discrepancy reported by different research groups. Nevertheless, the above findings, together with the recent identification of ST2 expression by oligodendrocytes (Table 2) indicate an important role for IL-33/ST2 signaling pathway in the myelination process during the CNS development, and also likely the repair phase in demyelinating diseases such as MS. Additionally, ST2 is shown to be expressed in small to medium-sized dorsal root ganglion (DRG) neurons and IL-33 induces Ca^2+^ influx into a subsets of neurons dissociated from the cervical DRG, suggesting IL-33/ST2 signaling excites sensory neurons (36).

The unique role of IL-33 in CNS physiological function *in vivo* has been reported using *Il33* gene knockout mice (21). Deficiency of *Il33* alters the expression of c-Fos proteins, an indicator of neuronal activities, in brain regions implicated in anxiety-related behaviors. These IL-33 deficient mice exhibit reduced anxiety-like behaviors, as well as deficits in social novelty recognition, despite intact sociability (21). The authors suggest that IL-33 may regulate the development and/or maturation of neuronal circuits, rather than control neuronal activities in adult mice. Indeed IL-33 mediated signaling is required in brain development (16). IL-33 deficient mice contain an excess number of excitatory synapse and show deficits in acoustic startle response, a sensorimotor reflex mediated by motor neurons in the brain stem and spinal cord. Furthermore, IL-33 produced by synapse-associated astrocytes is required for the development of normal synapse numbers and circuit function in the thalamus and spinal cord, signaling primarily through microglia cells under physiologic conditions to promote increased synaptic engulfment (16).

While the above findings reveal a key role for IL-33 during CNS development and activities in some brain regions, it is important to remember that the mechanisms of action of the IL-33/ST2 axis in the CNS are likely to be complicated and engage the multi-cell network consisting of neurons, oligodendrocytes, astrocytes, and microglia cells. The findings of IL-33 involvement in exciting sensory neurons, axonal myelination and synapse homeostasis also imply that dysregulated IL-33/ST2 signaling may lead to CNS neurological and behavioral disorders.

## Role of IL-33/ST2 axis in CNS disease

### Alzheimer's disease

AD is the most common form of dementia and its pathology can be characterized by extracellular amyloid deposits- made of Aβ peptides- and intracellular tau-based neurofibrillary tangles (NFTs) (48). Genetic studies have identified single nucleotide polymorphisms (SNPs) within *Il-33* to be associated with a decreased risk of developing AD in Caucasian (46) and Han Chinese populations (49, 50). It is not yet clear how the expression levels of IL-33 and ST2 in tissues correlate with AD. Chapuis observed a reduced level of IL-33 expression in the brains of AD patients (46). However, increased IL-33 and ST2 levels are found in proximity to amyloid plaques and NFTs in AD patients in comparison to healthy controls (51). Patients with mild cognitive impairment, who have an increased risk of AD development, also exhibit a significantly higher serum level of sST2 (52).

It remains debatable whether and how IL-33 affects AD. The induction of IL-33 production by β-amyloid peptide in astrocytes at the vicinity of plaques indicates IL-33 may contribute to AD pathogenesis as one of the inflammatory molecules (51). Indeed an effective treatment with a nanomaterial in *APP/PS1* transgenic mice, a transgenic animal model of amyloid deposition, shows improved learning and memory capability associated with decreased levels of several pro-inflammatory cytokines including IL-33 (53). Other investigators disagree and have presented research evidence to substantiate a potentially therapeutic role for IL-33 in AD (18, 46, 52). Systemic injection of rIL-33 in *APP/PS1* mice reverses synaptic plasticity impairment and memory deficits with reduced soluble Aβ levels and amyloid plaque deposition (52). This is likely mediated by promoting the recruitment of microglia and enhancing their Aβ phagocytic activity. Their subsequent experiments revealed that IL-33 polarizes microglia and macrophages toward an anti-inflammatory M2 phenotype, a well-recognized mechanism of IL-33 action in several immune-mediated diseases including spinal cord injury (12), asthma (54), and EAE (29). Such a beneficial role of IL-33 in AD is further supported in a study using aging mice. IL-33 expression in astrocytes is dramatically increased by up to 74% in aged mice, which appears to be critical for the repair of aged neurons (18). Conversely IL-33 deficient mice have an uncontrolled surge of neuronal aging due to failed repair at middle age and ultimately develop neurodegeneration and late-onset AD-like symptoms in old age (18). This is characterized by Tau deposition and heavy neuronal loss in both the cerebral cortex and the hippocampus and accompanied with cognition and memory impairment. These findings indicate a critical role for IL-33 in the maintenance and repair of aging and stressed neurons.

In summary, further investigation is required to determine whether and how the changed levels of IL-33, ST2 and/or sST2 in CNS tissues imply a specific role of IL-33/ST2 signaling pathway in AD. Evidences from *in vivo* research using animal models support a neuroprotective role by modulating neuroinflammation and promoting neuronal repair process, indicating a potential IL-33 based novel therapeutic strategy for AD patients.

### Multiple sclerosis

The involvement of IL-33 in the development of MS, a CNS inflammatory demyelinating autoimmune disease, has been supported by the increased expression of IL-33 and/or ST2 in the CNS lesions of MS patients (13, 19) and EAE mice (29, 55). In addition, effective treatment in EAE rats downregulates IL-33/ST2 expression (55). However, its precise function in disease development remains controversial (29, 32, 56). While Li et al. reported a detrimental effect of IL-33 treatment on EAE severity (56), others argue a protective role for the cytokine in attenuating EAE. The studies suggest IL-33 inhibits EAE development through mechanisms such as promoting type 2 T cell- and macrophage-mediated immune responses and inhibiting production of IL-17 and IFN-γ (11, 29). A more recent investigation suggests that a dramatic decrease of intracellular IL-33 is accompanied by increased extracellular IL-33 in the spinal cord of EAE mice (32). This subsequently promotes the expansion and function of ST2^+^ Treg cells in inhibiting CNS inflammation. Interestingly, IL-33 has been reported to regulate sex-dimorphic susceptibility of MS (57). Male-specific expression of IL-33 by mast cells expands ST2 positive (58) type 2 innate lymphoid cells (ILC2s) and drives a Th2 immune response in attenuating EAE clinical symptoms in mice. To date no data are available indicating a gender-based differential expression/production of IL-33 and/or ST2 in tissues of other sex-biased diseases. The involvement of mast cells and ILC2s as the mediating cells in the above study indicates that this special function of IL-33 is unlikely to be limited to CNS diseases. Thus, future studies in immune disorders with pronounced gender differences such as systemic lupus erythematosus and ankylosing spondylitis may provide improved knowledge of the molecular basis of sex-dimorphic disease.

Another emerging issue of recent debate is whether IL-33/ST2 plays an important role in remyelination, an essential CNS repair process in MS. This is of particular interest after the confirmation of ST2 expression on oligodendrocytes (12, 13). Despite the findings that rIL-33 reduces the proportion of myelinated axons in a rat myelinating culture (13), higher levels of IL-33 expression are observed in tissues with higher myelin content *in vivo* (12). rIL-33 also promotes the differentiation and maturation of rat oligodentrocytes in culture as shown with increased transcription of myelin genes and phosphorylation of p38MAPK, a signaling molecule involved in myelination (27). Furthermore, *in vivo* subcutaneous administration of Poly-IC results in greater recruitment of OPCs and enhances remyelination following gliotoxic injury with lysolecithin to the brain CC region (27). This is associated with increased expression of IL-33 in astrocytes and an upregulation of Arg and CD206 in macrophages (indicating they are type 2 like macrophages) in the local region.

Therefore, it is clear that current research findings support the involvement of IL-33/ST2 axis in MS development through modulating both the immune response and the CNS repair process. However, its precise function remains to be determined before any potential strategies of therapies can be developed for patients.

### Schizophrenia

Schizophrenia is a complex neuropsychiatric disorder which is characterized by a heterogeneous combination of symptoms such as hallucinations and delusions, social withdrawal as well as cognitive impairment. It affects approximately 1% of the worldwide population, with its pathogenesis yet to be fully characterized. Recent studies have shown that specific cytokines might be the neurobiological mediators underlying the pathology of the disease (59). Elevated levels of inflammatory cytokines such as IL-1β, IL-12, IFN-γ, and TNF-α are detected both in the brain and blood of schizophrenia patients (60). Furthermore, increased levels of serum IL-18 is positively associated with cognitive deficits in schizophrenia patients (61). To date very little is known about IL-33 in schizophrenia. However, the expression of IL-33 and ST2 in schizophrenia associated brain regions such as prefrontal cortex, basal ganglia, hippocampus, and amygdala (62–65) may suggest its involvement in disease development. Indeed polymorphism of IL-33 is associated with decreased susceptibility to schizophrenia within an Iranian population (66). Additionally, increased levels of serum IL-33 and sST2 correlate with improved cognitive performance in schizophrenia patients, although no difference of either molecules is observed between patients with schizophrenia and healthy controls (67).

**Figure 5:**
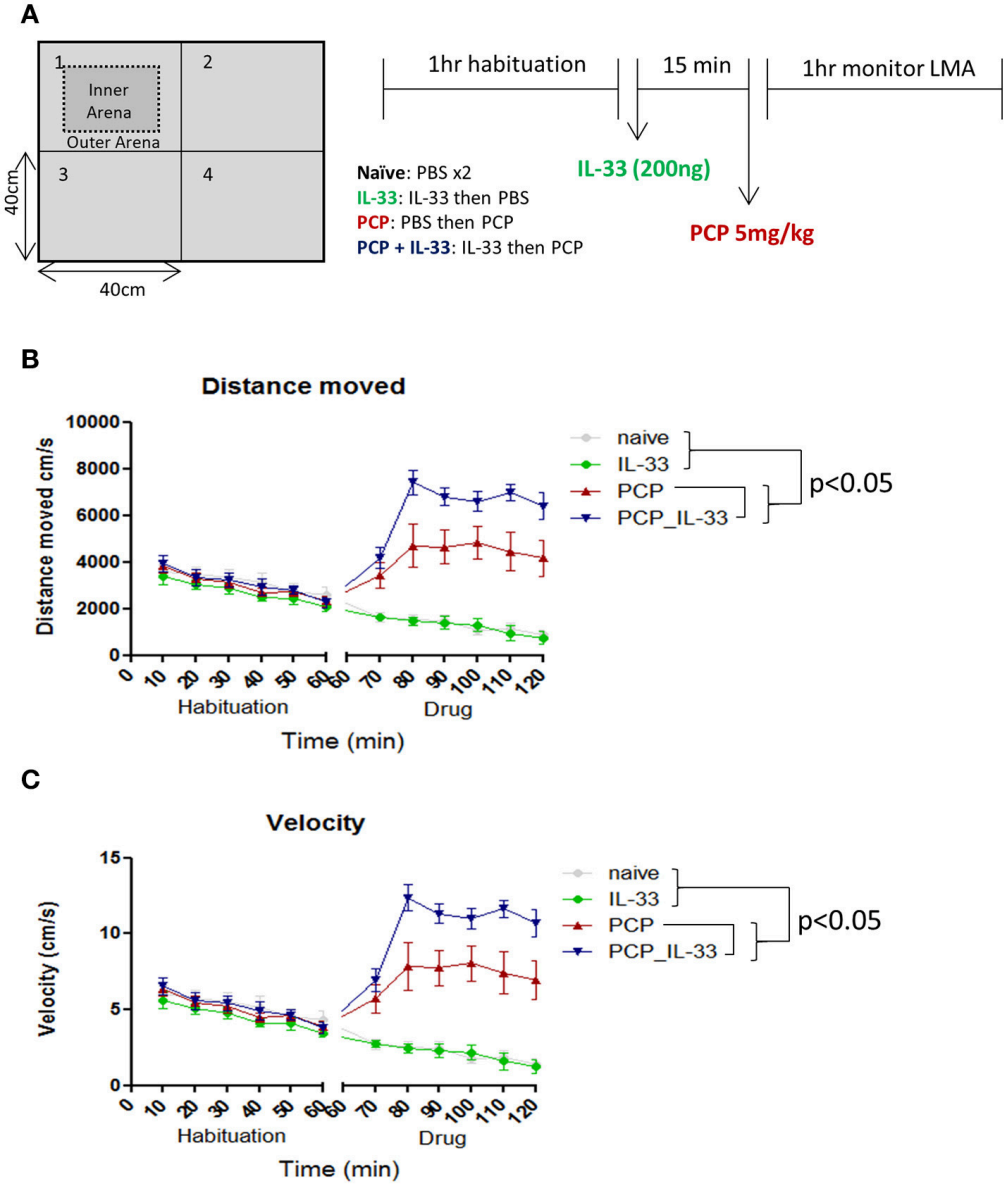
IL-33 enhances PCP-induced changes in locomotor activity. **(A)** C57BL/6 mice were first habituated inside a 40 × 40cm open field maze for 1 h before being taken out of the maze and injected intraperitoneally with vehicle control (PBS) or 200 ng IL-33. After 15 min mice received an i.p. injection of either PBS or PCP (5 mg/kg), and were then placed back into the same open field maze for 1 h. Their **(B)** distance moved and **(C)** velocity were tracked via overhead cameras and analyzed. Significant (*p* < 0.05) pairwise differences according to Tukey's *post hoc* test show PCP_IL-33 > PCP > naïve and IL-33 for both distance moved and velocity. LMA: locomotor activity.

Schizophrenia patients frequently exhibit behavioral abnormalities such as enhanced persistence of resting and active periods of locomotor activities (68). IL-33 deficient mice display multiple behavioral deficits such as reduced anxiety and impaired social recognition (21). However, these mice show no obvious changes in locomotor activities, with the exception of older mice (60 weeks old) at which point the activities are increased (18). Naïve adult age (8–10 weeks) mice receiving intraperitoneal injection of rIL-33 do not show any change in locomotor activity when analyzing parameters such as distance moved, velocity, rotations and meander in an open-field test (personal observation). Interestingly in a mouse model of schizophrenia induced by phencyclidine (PCP) (69–71), a non-competitive antagonist of the N-methyl-D-aspartate (NMDA) receptor (72, 73), rIL-33 dramatically increases the PCP-induced locomotor activity. This is characterized by increased animal moving distance and velocity in open-field tests relative to PCP mice (Figures 5A–C; *P* < 0.0001).

The effect of IL-33/ST2 signaling pathway on locomotor activity in both aging (18) and PCP treated mice, but not normal age naïve mice, is very interesting and important. Future studies should reveal whether the IL-33/ST2 signaling pathway only influences locomotor activity in individuals with specific CNS conditions. The underlying mechanism of altered locomotor activities by IL-33 remains unclear, however as an antagonist of NMDA receptors, IL-33 may act on glutamate synapses in a synergistic fashion similarly to IL-1β (74) and TNF-α (75). It is also possible that IL-33 may influence the secretion of glutamine synthetase by astrocytes. This would potentially have an impact on the ability of astrocytes to protect neurons from excitotoxicity which has been shown before with IL-1β and TNF-α (76).

### CNS injury

CNS traumatic or ischemic injury is one of the leading causes of death and permanent disability worldwide. Neuroinflammation, often characterized by type 1 or type 2 immune responses (although an oversimplified description, but helpful in our understanding of the immune responses in diseases), is a prominent feature of CNS injury and highly influences CNS repair and thus the injury recovery process (77–80). As a nuclear alarmin molecule released by damaged dying cells (6) and as an important immunomodulatory cytokine, IL-33 plays an important role in the pathophysiology of CNS injury.

An association between IL-33/ST2 signaling pathway and CNS ischaemic injury has been highlighted by the findings of elevated level of sST2 in the plasma of stroke patients and its correlation with a worsened clinical outcome (81). Significantly increased level of serum IL-33 is also detected in patients with acute ischemic stroke (AIS) compared with healthy controls (82), and is shown to be a novel diagnostic and prognostic biomarker for AIS. Patients with higher IL-33 demonstrate a favorable outcome 3 months after the stroke. In a mouse model of stroke, the expression of IL-33 by oligodendrocytes and astrocytes is rapidly increased together with an upregulation of ST2 on microglia after inducing ischemic brain injury (28). Increased ischemic lesion size and long term behavioral deficits are observed in ST2 deficient mice in comparison to the wild type controls indicating a possible protective role of IL-33 in stroke (28). Indeed administration of rIL-33 locally (28, 83) or peripherally (81) reduces stroke-induced CNS damage and ameliorates neurological deficits via inducing anti-inflammatory responses systemically and M2 type macrophages and microglia in the CNS. This occurs at least partially in an IL-4-dependent manner (81). Meanwhile infusion of rIL-33 intracerebroventricularly protects mice from ischemic injury by inducing a switch from Th1 to Th2 and suppressing Th17 responses (83), or by potentiating the expression of IL-10 and other M2 genes in microglia (28). Indeed mice deficient in ST2 gene have impaired expression of M2 polarizing markers (28).

The IL-33/ST2 signaling pathway is also closely involved in the neuroinflammatory response following CNS traumatic injury. Significantly increased levels of IL-33, likely released by oligodendrocytes (12) and astrocytes (31), are detected in injured spinal cord segments (12, 31) and in the cerebrospinal fluid (CSF) (12). Gadani et al. further discovered that the baseline expression level of *St2* transcript by astrocytes is low but significantly elevated after injury. At the same time its expression level on microglia is shifted from high at baseline to low after injury. From these data the authors concluded that astrocytes but not microglia are likely to be the primary target cells of IL-33 mediating the neuroinflammation after injury. It also confirms the dynamic change of IL-33 expression in CNS tissues under diseased conditions (12). Mice with spinal cord injury exhibit significantly reduced tissue damage, demyelination and astrogliosis after treatment with rIL-33 (31). This is associated with suppressed pro-inflammatory immune responses and biased anti-inflammatory M2 microglia/macrophages and Treg cells both locally in the spinal cord and systemically (31). On the contrary, mice deficient of IL-33 gene develop increased lesion size together with significantly decreased number of M2 microglia and macrophages (12). The investigators further demonstrated that IL-33 released from damaged oligodendrocytes upon injury orchestrates the production of various chemokines such as CCL2 and CXCL2 by astrocytes, resulting in the recruitment of monocytes into the CNS. As a potent activator of ST2 expressing ILC2s (39, 58, 84), IL-33 released into the CSF immediately after injury also mediates CNS inflammation through the newly discovered meningeal ILC2s (85). IL-33 therefore is released as an alarmin after injury to orchestrate the activations of both CNS cells and immune cells with the aim to promote recovery (12).

Taken together, in both ischemic and traumatic CNS injury, IL-33 signaling appears to be beneficial. IL-33 released by damaged oligodendrocytes, astrocytes and possibly other cells at the injury site coordinates the immune responses mediated by CNS resident cells, meningeal ILC2s and infiltrating immune cells. This ultimately promotes a type 2-like neuroinflammation and confers protection from tissue damage and neurological deficit.

### Pain

Given the prevalence of chronic pain and the fact that current treatment only provides moderate degrees of relief, it is both challenging and important to understand the molecular mechanisms of pain. Over the years astrocytes and microglia cells have emerged as key contributors to the pathological development of pain (86) through releasing a number of important molecules such as IL-33.

IL-33 was first identified as a key mediator of inflammatory hypernociception in an animal model of arthritis (87), and in carrageenan-induced inflammatory pain likely through triggering the production of inflammatory mediators such as TNF-α (88). In a more recent study, Zarpelon et al. show that intrathecal injection of IL-33 induces hyperalgesia in naïve mice and enhances hyperalgesia caused by chronic constriction injury (CCI) (34). Furthermore, hyperalgesia is reduced in mice deficient of *St2* gene or treated with IL-33 decoy receptor sST2 suggesting IL-33 has an important role in neuropathic pain. The group further demonstrated IL-33 is mainly produced by spinal cord oligodendrocytes after CCI which subsequently acts on astrocytes and microglia. Furthermore, IL-33-mediated hyperalgesia is dependent on a reciprocal relationship with TNF-α and IL-1β. This mechanism of action of IL-33 is supported by findings from a rat model of radicular pain (35). Non-compressive lumbar disc herniation induces expression of IL-33 and ST2 in the spinal cord which subsequently mediates the development and progression of radicular pain through modulating TNF-α, IL-1β, and COX-2. The pathogenic contribution of IL-33 toward pain is further confirmed in a spared nerve injury induced neuropathic pain model (33). Blocking of IL-33/ST2 signaling with ST2 neutralizing antibody or *St2* gene depletion significantly attenuates mechanical and cold allodynia. Liu et al. reported that IL-33 induced nociceptive behavior involves the spinal NMDAR and is mediated through activation of the astroglial JAK2–STAT3 cascade and the neuronal CaMKII–CREB cascade (33).

Confirming the pathological contribution of IL-33/ST2 signaling pathway in pain development, effective reduction of CCI induced pain by different analgesics is shown to be mediated through inhibition of the IL-33/ST2 signaling pathway (89–91). This also agrees with a report by Han et al. suggesting electro acupuncture analgesia alleviates formalin-induced inflammatory pain in mice, at least partially, through inhibition of spinal IL-33/ST2 signaling and the downstream ERK and JNK pathways (92).

Based on the above findings, it is likely that IL-33/ST2 induces and/or augments pain in the nervous system through potentiating TNF-α and IL-1β mediated inflammation. Thus, future therapeutic strategies for patients with pain may be developed by targeting the IL-33/ST2 signaling pathway along with current treatments.

### CNS parasitic infection

IL-33 is important in limiting protozoan parasite infections systemically (93, 94), with several studies particularly focused on its role in neuropathology induced by parasite infections. Toxoplasma gondii (*T. gondii*) is an obligate, intracellular parasite which is able to persist, often asymptomatically, for a lifetime within the CNS of immunocompetent hosts. *St2* mRNA is upregulated in the brain of mice infected with *T. gondii* (95). ST2 deficient mice (*T1/St2*^−/−^) demonstrate an increased susceptibility to cerebral infection with an increased parasite burden and more severe encephalitis which is associated with greater cerebral expression of iNOS, TNF-α and IFN-γ (95). This indicates a possible protective role for IL-33/ST2 signaling in *T. gondii* infection.

A specific role of IL-33 has been investigated in cerebral malaria which is the most severe form of neurological complications associated with *Plasmodium falciparum* infection. It results in long term cognitive deficits, behavioral difficulties, epilepsy, coma and, in many cases, death (96). Increased IL-33 expression is shown in the brains of experimental cerebral malaria (ECM) mice induced by murine *Plasmodium berghei* ANKA (*Pb*A) (25, 97), which is likely expressed by astrocytes and oligodendrocytes (25). Meanwhile, ST2 deficient mice do not exhibit ECM associated neurological signs or associated cognitive deficits unlike their wild type counterparts despite similar levels of parasitaemia and parasite load (25, 97). There appears to be reduced neuroinflammation together with a reduction in activated CD4^+^ and CD8^+^ T cells and pro-inflammatory cytokines and chemokine levels within the brain parenchyma of ST2 deficient mice. A potential feedback loop has been further suggested between microglia and oligodendrocytes in exacerbating neuroinflammation by the initial infection. IL-33 stimulates IL-1β production by microglia, which in turn induces IL-33 expression by oligodendrocytes (25). These results suggest IL-33/ST2 signaling plays a role in promoting the effector and cytotoxic T cell responses and enhancing the CNS resident immune response, which combined contributes to the neuropathology and cognitive deficits associated with ECM. Paradoxically, administration of IL-33 in the early stages of ECM improves animal survival time with reduced weight loss and clinical score, but not from malaria-induced hyperparasitemia and death compared to control mice (39). The study provided further evidence of a protective immune response via IL-33-induced ILC2s, M2 macrophages, and T regs. It is not known what has caused the discrepancy between the above findings, the data may highlight the difference between research approaches using gene modified animals and exogenous administration of reagents.

Thus, while the study by Jones et al. illustrates a protective role for IL-33 in *T. gondii* infection (95), the precise pathophysiological function of IL-33/ST2 in ECM remains to be determined.

### Glioma

The role of IL-33 in tumor progression has been illustrated elsewhere, with overexpression of IL-33 identified as a diagnostic and prognostic marker for several types of cancer (98–101). Within the CNS, IL-33 is abundantly expressed by rat glioma cells together with its receptor ST2 (102). In glioma patients, increased expression of IL-33 (103, 104) and ST2 (104) is detected in tumor tissues albeit heterogeneously, compared with normal brain tissue (103). Zhang et al. also reported higher expression of *Il33* mRNA in glioma tissues than in normal brain tissue, and that the level correlates with a shorter progression-free survival and overall survival than those with low expression (105). Furthermore, while there is no differential IL-33 protein expression by tumor grade, elevated levels of IL-33 protein, and mRNA are associated with inferior survival in patients with recurrent glioblastomas. All of these data suggest a pivotal role for IL-33 in the disease pathogenesis and as a potential biomarker for prognosis of human gliomas.

It has become clear that the IL-33/ST2 signaling pathway facilitates glioma cell proliferation and migration, as treatment of the cells with IL-33 shRNA or ST2 shRNA reduces cell growth and colony formation in culture and reduces tumor volume *in vivo* (102). Further evidence indicates that the involvement of IL-33 in glioma cell invasion and migration is through upregulation of MMP2 and MMP9 via the ST2-NF-κB signaling pathway (104).

Thus, IL-33 may become a useful biomarker in predicting prognosis in glioma patients, and a novel therapeutic target for glioma treatment.

### Other CNS diseases

Emerging evidence suggests that the role of IL-33 is not limited to the diseases highlighted in this paper (Table 3) and is expanding to other CNS diseases. Expression level of IL-33 is significantly reduced while sST2 increased in the serum samples of amyotropic lateral sclerosis patients (113). In a rat model of subarachnoid hemorrhage (SAH), expression of IL-33 in the cerebral cortex after injury is markedly elevated in the SAH together with IL-1β and TNF-a (26). IL-33 signaling pathway is also shown to be essential in attenuating viral-induced encephalitis development by downregulating iNOS expression in the CNS (110). Although it is not changed in autism spectrum disorder patients (107), increased plasma level of IL-33 is also observed in bipolar disorder patients (114). While it will take time to appreciate the specific and important function of IL-33/ST2 in various CNS disorders, future research progress will provide new knowledge and lead to improved diagnosis and therapies for patients.

**Table 3:** Role of IL-33/ST2 axis in neurological disease.

**Disease**	**Role of IL-33**	**References**
Alzheimer disease (AD)	• Reduced IL-33 expression in AD brain; *Il33* genetic variants associate with a decrease risk in AD	(46)
	• Increased IL-33 and ST2 in AD brains, Aβ induces IL-33 production by astrocytes to mediate AD pathogenesis	(51)
	• Increased sST2 in serum of AD patients; IL-33 reduces Aβ levels and amyloid plagues and reverses synaptic plasticity impairment and memory deficit in a mouse model	(52)
	• Effective treatment of *APP/PS1* mice with improved learning and memory capability is associated with reduced level of IL-33	(53)
	• IL-33 expression in astrocytes increases with age and IL-33^−/−^ aged mice display AD-like symptoms with tau abnormality and neurodegeneration	(18)
Multiple sclerosis (MS)	• Elevated IL-33 protein, mRNA levels in NAWM and plaque areas, and in plasma and PBMCs	(19, 106)
	• Enhanced expression of IL-33 and ST2 in MS CNS lesion	(13)
	• mRNA of IL-33 and ST2 increased in spinal cord of EAE rats	(55)
	• IL-33 blockade suppresses EAE development in mice	(56)
	• IL-33 attenuates EAE by inducing type 2 immune response	(29)
	• IL-33 released by astrocytes and neurons suppresses EAE	(32)
	• Male-specific IL-33 attenuates EAE through ILC2s and mast cells	(57)
Pain	• IL-33 is a key mediator of inflammatory hyper nociception	(87)
	• IL-33 mediates carrageenan-induced inflammatory pain via trigging TNF-α	(88)
	• IL-33 induces hyperalgesia in naïve mice and enhanced hyperalgesia in a mouse model of chronic constriction injury via reciprocal relationship with TNF-α and IL-1β	(34)
	• IL-33 inhibits electroacupuncture analgesia in formalin mice	(92)
	• Spinal IL-33 and ST2 contribute to bone cancer induced pain in mice	(30)
	• IL-33 contributes to neuropathic pain through activating astroglial and neuronal cascades, and up-regulate NMDA	(33)
	• IL-33 induced by non-compressive lumber disk herniation in rat mediates pain through TNF-α, IL-1β and COX-2	(35)
Psychiatric disorders	• IL-33 polymorphism associates with decreased susceptibility to schizophrenia	(66)
	• Serum levels of IL-33 and sST2 positively correlate with cognitive performance in schizophrenia patients, but with no difference between patients and controls	(67)
	• IL-33^−/−^ mice display multiple behavioral deficits, e.g., reduced anxiety and impaired social recognition	(21)
	• IL-33^−/−^ aged mice exhibit increased locomotor activities	(18)
	• IL-33, sST2 and IL-1β plasma levels are not changed in ASD patients	(107)
	• Reduced plasma IL-33 in ASD patients	(108)
Stroke	• sST2 is increased in plasma of stroke patients, IL-33 protects mice against stroke through IL-4	(81)
	• IL-33 ameliorates brain injury in stroke model through promoting Th2 and suppressing Th17 responses	(83)
	• Increased serum IL-33 in acute ischemic stroke patients	(82)
	• IL-33/ST2 dependent microglial response limits acute ischemic brain injury	(28)
Traumatic CNS injury	• Oligodendrocyte-derived IL-33 induces M2 genes and aids the recovery of optic nerve injury in mice	(12)
	• IL-33 reduces secondary injury and improves recovery in a mouse contusion injury model through inducing Th2 and Treg responses	(31)
	• IL-33 improves spinal cord injury via activating meningeal ILC2s to produce type 2 cytokines	(85)
Cerebral malaria	• IL-33 treatment reduces parasitaemia at early phase of infection through inducing type 2 immune responses	(39)
	• ST2 deficient mice survived longer than WT after infection	(97)
CNS encephalitis	• Levels of IL-33 protein and mRNA increased in the serum and CSF of neuro-Behcet's disease patients	(109)
	• IL-33 attenuates viral induced encephalitis by downregulating IFN-γ and NO production	(110)
CNS Toxoplasmosis	• ST2 Levels increased in brain of infected mice, ST2 deficient mice had increased susceptibility, parasite burden and encephalitis to cerebral infection	(95)
CNS hemorrhage	• IL-33 encoding gene *Dvs 27* is active after experimental subarachnoid hemorrhage	(111)
	• *Il33* mRNA elevated in cerebral cortex in subarachnoid hemorrhage	(26)
	• IL-33 has neuroprotective effects in intracerebral hemorrhage in mouse	(112)

## Conclusions

The broad expression of IL-33 and ST2 in different CNS regions and cells, in addition to many well-documented immune cells, suggests the involvement of IL-33/ST2 signaling pathway in the complex network of multi-cell interaction between the immune and nervous systems in CNS health and disease. Scientific advancements during the last decade have provided some initial insights into the diverse and pleiotropic role IL-33 plays in certain aspects of CNS function and disease through interactions between glia, neuron, and immune cells. However, as the CNS is the most complex organ, and human disease often displays multiple layers of pathophysiology, the challenge now is to determine in more detail the intensive and dynamic communications among the CNS resident and infiltrating cells. This would enhance our understanding of the important roles the IL-33/ST2 signaling pathway plays in CNS homoeostasis and neurological disorders. These findings will advance our diagnosis of CNS diseases, and develop novel effective therapeutics for patients.

## Ethics statement

Tissues in Figures 1–4 were collected from naïve C57BL/6 mice at 8–10 weeks of age. All animal care and experimental procedures were conducted in accordance with relevant guidelines and regulations with the approval of the University of Strathclyde Animal Welfare and Ethical Review Body (AWERB), under UK Home Office regulations [Animals (Scientific Procedures) Act 1986, UK]. Animals are housed according to the Home Office Code of Practice for the housing and care of animals bred, supplied or used for scientific purposes.

## Author contributions

KF-C, MB, SH, and DA performed the experiments and analyzed the data. MB, CW, SH, DA, H-RJ and KF-C made contribution to data interpretation, figure and manuscript preparation. MB and H-RJ revised and finalized the files for submission.

### Conflict of interest statement

The authors declare that the research was conducted in the absence of any commercial or financial relationships that could be construed as a potential conflict of interest.
